# A temporal dialogue between the circadian clock and chronic inflammatory diseases

**DOI:** 10.1242/dmm.052749

**Published:** 2026-05-07

**Authors:** Siyu Chen, David Ray

**Affiliations:** ^1^ NIHR Oxford Health Biomedical Research Centre, Warneford Hospital, Warneford Lane, Oxford OX3 7JX, UK; ^2^ NIHR Oxford Biomedical Research Centre, John Radcliffe Hospital, Headley Way, Headington, Oxford OX3 9DU, UK; ^3^ Oxford Centre for Diabetes, Endocrinology and Metabolism (OCDEM), Radcliffe Department of Medicine, University of Oxford, Churchill Drive, Oxford OX3 7LE, UK; ^4^ Kavli Institute for Nanoscience Discovery (INsD), University of Oxford, Sherrington Road, Oxford OX1 3QU, UK

**Keywords:** Circadian clock, Chronic inflammatory diseases, Immunity, Inflammation, Metabolism

## Abstract

The circadian clock is an intrinsic molecular system that synchronises biological processes with daily environmental cycles. Under physiological conditions, cell-intrinsic clocks and systemic cues together regulate the circadian rhythmicity of immune activity, limiting immune responses to the appropriate time and intensity for optimal energy allocation and fitness. In chronic inflammation, by contrast, persistent and profound circadian alterations may cause a pro-inflammatory shift of homeostasis and hinder resolution. Circadian-based therapeutic strategies are emerging as promising approaches to overcome the limitations of conventional anti-inflammatory therapeutics and relieve treatment burden. This Review examines the bi-directional relationship between circadian regulation and chronic inflammation across immune-mediated, metabolic and infectious conditions. Circadian rhythms shape the timing, severity and tissue specificity of inflammatory responses, while inflammatory signals from diverse pathological settings converge on shared transcriptional nodes that interface with the clock, altering temporal organisation across multiple systems. We further highlight key future directions, including defining the molecular links between the circadian clock, inflammation and metabolism for precise target identification, restoring the intrinsic capacity for temporal homeostatic regulation through personalised circadian medicine, and integrating behavioural and environmental factors into the current framework. Together, they represent a path towards more precise, preventive and holistic management of chronic inflammatory diseases.

## Teaser

**Summary:** This Review provides an integrative understanding of crosstalk between circadian clocks and chronic inflammatory diseases, highlighting the advantages of circadian therapies and future directions towards precise, preventive and holistic healthcare.

## Introduction

Most life on Earth has evolved physiological and behavioural rhythms with a period of approximately 24 h, to anticipate and adapt to daily environmental changes (Bhadra et al., 2017). These near-24-h (circadian) rhythms persist even in the absence of environmental changes, indicating control by an endogenous timing system. This intrinsic timing system, the circadian clock, generates rhythmic biological processes and is continually entrained by environmental signals to align physiology with the 24-h day, especially light cycles (Bhadra et al., 2017).

At the organism level, the master circadian pacemaker of mammals resides in the suprachiasmatic nucleus (SCN) of the hypothalamus. The SCN receives light signals from the retina via the retinohypothalamic tract (Moore and Lenn, 1972). Early lesion studies showed that disruption of the SCN abolishes behavioural and endocrine rhythms in rats (Moore and Eichler, 1972). The pacemaker role of SCN was further proved by transplantation experiments in hamsters, in which grafting SCN tissue restored rhythmicity in arrhythmic hosts (Ralph et al., 1990).

It has emerged that all cells have intrinsic timekeeping properties and are entrained to the light−dark cycles by systemic cues from the SCN. At the cellular level, circadian timekeeping is driven by cell-autonomous molecular clocks composed of interlocking transcription−translation feedback loops (TTFLs). In these loops, clock components activate transcription of genes whose products subsequently inhibit their own expression, generating approximately 24-h oscillations in gene expression and downstream physiological processes (Patke et al., 2020). Similar cell-autonomous clocks exist in almost all mammalian nucleated cells and are organised across tissues by the SCN (Bechtel, 2024). While the SCN is primarily entrained by the light−dark cycle and coordinates peripheral clocks through systemic signals, peripheral clocks also respond to non-photic cues, such as temperature, nutrition, physical activity and disease states (Bechtel, 2024). This multi-layered network ensures both systemic synchronisation and tissue-specific circadian activities.

The mammalian immune system is a complex and dynamic network distributed across the body to maintain homeostasis and protect the body from pathogens, tissue damage, and malignancies (Poon and Farber, 2020). Immune responses are energetically costly and can cause tissue damage when excessive or prolonged (Man et al., 2016). Therefore, immune responses must be precisely regulated to ensure they can be initiated rapidly and effectively when needed, while their costs and collateral damage remain controlled. The circadian clock contributes to this control by anticipating environmental changes to prepare the immune system for upcoming challenges and by time-gating immune responses to optimal timing and intensity (Man et al., 2016).

Accordingly, nearly all aspects of immunity show circadian oscillations, from the steady-state immune surveillance to innate and adaptive immune responses (Labrecque and Cermakian, 2015). Evidence for circadian regulation of immunity dates back to the 1950s, when Halberg and colleagues reported daily rhythms in circulating eosinophils and endotoxin susceptibility in mice (Halberg and Visscher, 1950; Halberg et al., 1960). Subsequent studies have identified circadian regulation of numerous immune processes (as reviewed by Scheiermann et al., 2013; Wang et al., 2022). Some key processes include leukocyte tissue recruitment to tissues in mice (Scheiermann et al., 2012), pattern recognition receptor signalling in mouse immune cells (Silver et al., 2018), mouse peritoneal macrophage phagocytosis (Hayashi et al., 2007) and cytokine secretion in mouse spleen cells (Keller et al., 2009). Large cohort studies in humans similarly reveal circadian variation in peripheral blood lymphocyte and neutrophil counts (Wyse et al., 2021), and antiviral cytokine production (Diallo et al., 2021). Functionally, rhythmic immune activities are temporally partitioned, with increased immune sensitivity and surveillance in the active phase corresponding to higher risks of injury and infection, and enhanced tissue repair and inflammation resolution during the rest phase (Curtis et al., 2014).

Circadian control of immunity arises from both immune cell intrinsic clocks and rhythmic cues from their surrounding microenvironments (Man et al., 2016). Cell-autonomous clocks regulate daily cycles of clock-controlled genes (CCGs) and cellular activity, while systemic neuroendocrine and hormonal signals, such as sympathetic nervous system and glucocorticoids, further shape immune rhythms (Man et al., 2016; Méndez-Ferrer et al., 2008; Balsalobre et al., 2000). The relative contribution of cell-autonomous and systemic mechanisms differs between immune compartments.

The cell-intrinsic clock is predominant in time-gating innate immune cell activity, such as mouse monocyte trafficking (Nguyen et al., 2013), and macrophage inflammatory responses and phagocytosis (Kitchen et al., 2020; Keller et al., 2009). By contrast, adaptive immune responses appear less dependent on the cell-intrinsic clock, as the deletion of the core clock gene *Bmal1* did not significantly alter the differentiation and function of mouse T and B cells, suggesting stronger regulation by rhythmic signals from antigen-presenting cells, non-immune cells, or rhythmic circulating cytokines and hormones (Hemmers and Rudensky, 2015). This difference likely reflects the distinct timescales of immune responses: innate responses occur rapidly within hours (Marshall et al., 2018), aligning well within the period length of circadian oscillators, whereas adaptive immune responses, such as thymic T-cell selection unfold over days to weeks (Yates, 2014).

Circadian regulation of immunity provides an important foundation for understanding inflammatory disease. In acute inflammation, inflammatory signalling can transiently suppress clock gene expression, releasing immune responses from circadian constraint to facilitate pathogen clearance or tissue repair (Haimovich et al., 2010; Okada et al., 2008; Haspel et al., 2014; Man et al., 2016). By contrast, persistent circadian disruption in chronic inflammation may shift immune homeostasis towards sustained pro-inflammatory states and hinder disease resolution. Consistent with this idea, circadian dysregulation has been linked to multiple chronic inflammatory conditions in humans and animal models, including – but not limited to – asthma (Huang et al., 2025), the progression of rheumatoid arthritis (Wilantri et al., 2023), type 2 diabetes (Rivera-Alvarez et al., 2025) and *Helicobacter pylori* infection (Li et al., 2019). Despite these advances, an integrative understanding that connects these different disease subsets remains incomplete.

Recent advances in chronobiology, systems immunology, and human and animal studies reveal extensive circadian control of immune, metabolic and inflammatory pathways. Meanwhile, the global rise of chronic inflammatory diseases is placing growing strain on both patient quality of life and healthcare systems. Together, these trends highlight the urgent need for a framework that integrates circadian biology with the complex, multi-system nature of chronic inflammatory diseases to better understand their mechanisms and improve therapeutic strategies.

In this Review, we synthesise current evidence and present a model illustrating how the circadian clock interacts with inflammation and its interconnected processes, such as metabolism, to shape the pathophysiology of chronic inflammatory diseases. We further extend the discussion to clinical application to examine current circadian-based therapeutic strategies. Finally, we highlight key questions that may guide the next phase of research in this rapidly evolving field.

## The circadian clock machinery

**Fig. 1.**
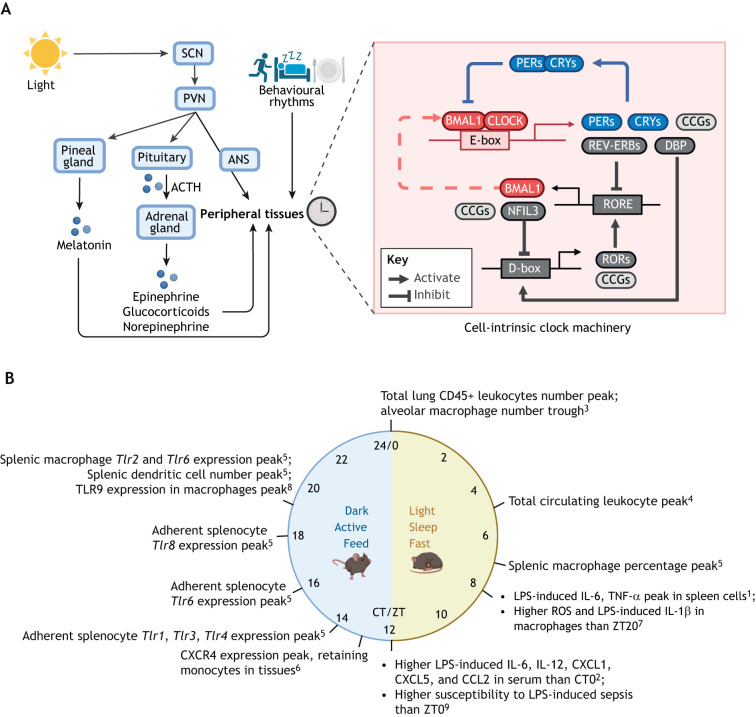
**The circadian clock machinery and circadian regulation of immune function.** (A) Left: Light input through the retina entrains the suprachiasmatic nucleus (SCN), which conveys rhythmic signals to the paraventricular nucleus (PVN). The PVN regulates (1) circadian patterns of hormone secretion through the hypothalamic−pituitary−adrenal (HPA) axis, (2) autonomous nervous system (ANS) outflow to peripheral organs and (3) pineal melatonin secretion. These signals, together with behavioural rhythms (sleep−wake cycles, activities and nutrition), regulate peripheral clocks across organs. Right: The cell-intrinsic circadian machinery. BMAL1 and CLOCK drive gene expression of their own repressor proteins PER and CRY, as well as of REV-ERBs and DBP. REV-ERBs compete with RORs in binding to ROR-response elements (ROREs), to inhibit or activate expression of BMAL1 and NFIL3. NFIL3 and DBP inhibit or activate D-box mediated expression of RORs. The positive and negative arms of the core transcription−translation feedback loops (TTFLs) are coloured in red and blue, respectively. Additional accessory loops are indicated in grey. ACTH, adrenocorticotropic hormone; CCGs, clock-controlled genes. (B) Peaks of circadian rhythmic immune processes in mice at steady-state unless otherwise stated. Superscript numbers 1-9 indicate references ^1^Keller et al., 2009; ^2^Gibbs et al., 2012; ^3^Haspel et al., 2014; ^4^Scheiermann et al., 2012; ^5^Silver et al., 2018; ^6^Chong et al., 2016; ^7^Early et al., 2018; ^8^Silver et al., 2012; ^9^Curtis et al., 2015. CT/ZT, circadian time/zeitgeber time; IL, interleukin; LPS, lipopolysaccharide; TLR, Toll-like receptor. Created in BioRender by Ray, D., 2026. https://BioRender.com/m8l7zdh. This figure was sublicensed under CC-BY 4.0 terms. Created in https://BioRender.com.

At the cellular level, the circadian clock machinery is formed by several intertwined TTFLs (Patke et al., 2020). The core TTFL consists of BMAL1 and CLOCK, i.e. basic helix-loop-helix (bHLH) and Per-Arnt-Sim (PAS) motif (bHLH-PAS) transcription factors, as the positive arm. These two proteins heterodimerise and activate the expression of the period circadian regulator (*Per*) and cryptochrome circadian regulator (*Cry*) genes (*Per1, Per2, Per3* and *Cry1, Cry2*, respectively), encoding PER and CRY proteins (PERs and CRYs, respectively) that form the negative arm of TTFL (Fig. 1A). PER and CRY form a complex and translocate into the nucleus, where they inhibit CLOCK/BMAL1-mediated transcription, including of their own genes. PER proteins are primarily phosphorylated by casein kinase 1 and casein kinase 2 (CK1 and CK2, respectively) and cyclin-dependent kinase 5 (CDK5), while CRYs are phosphorylated by adenosine monophosphate (AMP)-activated protein kinase (Brenna and Albrecht, 2020). Phosphorylation promotes the complex formation between PER and CRY (PER−CRY) and its nuclear translocation, while also making them targets of E3 ubiquitin ligases and, thereby, susceptible to proteasomal degradation. As levels of PER and CRY decline, repression of CLOCK–BMAL1 is relieved, allowing their transcriptional activity to resume and a new cycle to begin (Fig. 1A) (Patke et al., 2020). In addition to the core TTFL, BMAL1 and CLOCK also activate the expression of the heme-binding transcriptional repressor proteins NR1D1 and NR1D2 (also known as REV-ERBα and REV-ERBβ, respectively; hereafter referred to as REV-ERBα/β) and D-box-binding PAR bZIP transcription factor (DBP) that form two additional accessory regulatory loops (Fig. 1A) (Kim et al., 2022). In the first accessory regulatory loop, REV-ERBs and retinoic acid receptor (RAR)-related orphan nuclear receptors (RORs) that function as transcriptional activators, compete at the same REV-ERB and/or ROR response element (RORE), where REV-ERBs inhibit and RORs activate the expression of circadian clock genes, including BMAL1 and NFIL3 (also known as E4BP4), the latter acting as a transcriptional repressor binding the D-box motif (Preitner et al., 2002; Sato et al., 2004). In the second accessory regulatory loop, NFIL3 inhibits the expression of genes containing a D-box in their promoters, including those of RORs (Gachon et al., 2004), whereas DBP acts antagonistically to activate them (Gachon et al., 2004). Together, these multiple TTFLs form a robustly oscillating clock machinery that generates the rhythmic expression of numerous CCGs and, thereby, time-gate downstream biological processes to ensure they occur in the optimal temporal order and timing (Fig. 1A).

The cell-intrinsic clock machinery, together with environmental cues and the systemic neurohumoral signals, generates a wide range of circadian rhythmic immune processes (Fig. 1B). Cytokine secretion is one of the clock-controlled processes profoundly regulating immune function. In healthy diurnal humans, IL-6 shows a strong 24-h rhythm with a peak in the early morning (Nilsonne et al., 2016), whereas T-cell cytokines IFN γ and IL-10 show a slightly earlier peak around 03:00 during nighttime sleep (Zeng et al., 2024). In mice, endotoxin challenges reveal gated immune responses: *in vitro* in spleen cells, lipopolysaccharide (LPS)-induces peaks of IL-6 and TNF-α at the late sleep phase (Keller et al., 2009); *in vivo*, LPS-induced IL-6, IL-12, CXCL1, CCL2 and CCL5 levels in serum are significantly higher when LPS was given at the onset of the active phase rather than the rest phase (Gibbs et al., 2012), whereas serum levels of TNF-α are relatively consistent across a day. During chronic inflammation, rhythms persist – but with altered amplitude or phase shifts. For example, rheumatoid arthritis (RA) patients show a pronounced nocturnal rise in IL-6 levels, with a notably increased peak at ∼07:00 when compared with healthy individuals (Cutolo et al., 2008). The circadian pattern of cytokine secretion is also perturbed by circadian misalignment as, night-shift schedules for humans decreases mean levels of TNF-α and shift the timing of the expression peak of IL-6, a cytokine whose levels progressively increase with the duration of being awake (Liu et al., 2021). Additionally, levels of circulating leukocytes (Scheiermann et al., 2012), lymphocyte trafficking and tissue homing (Chong et al., 2016), pattern-recognition signalling (Silver et al., 2012, 2018) and animal susceptibility to LPS-induced sepsis (Curtis et al., 2015) also peak at different times of the day (Fig. 1B).

## The circadian clock regulates chronic inflammatory diseases

Many, if not all, chronic diseases have an inflammatory dimension. Given the variability in how terms such as ‘chronic inflammatory diseases’ and ‘chronic diseases’ are used, it is useful to clarify their definitions within the context of this Review before delving into details (Box 1).

Box 1. Chronic inflammatory diseases discussed in this Review In its core definition, ‘chronic inflammatory diseases’ often refers to “a spectrum of non-communicable immune-mediated diseases with a polygenic mode of inheritance, which may affect almost all organ systems” (Schultze and Rosenstiel, 2018). However, it is equally important to recognise that chronic inflammation also underlies many other conditions that are not primarily driven by immune dysfunction. For example, the four main types of chronic disease defined by the World Health Organization – i.e. cardiovascular disease, cancer, chronic respiratory disease and diabetes – together account for nearly three-quarters of global non-pandemic mortality (see https://www.who.int/news-room/fact-sheets/detail/noncommunicable-diseases), and each type exhibits a significant inflammatory component. Therefore, although cancer will not be a major focus in this Review – both for brevity and because only certain cancers are consistently considered chronic illnesses (Bernell and Howard, 2016) – it would be of great clinical importance to discuss other disease groups in this Review. Finally, while the inclusion of chronic infectious diseases within this framework remains debatable (Bernell and Howard, 2016), we do cover them here for conceptual completeness, because they represent a distinct etiology of chronic inflammation. Therefore, we consider chronic inflammatory diseases within a broad sense, encompassing three major categories:

(1) Immune-mediated inflammatory diseases (IMIDs), such as rheumatoid arthritis, asthma, chronic obstructive pulmonary disease (COPD), pulmonary fibrosis (PF) and inflammatory bowel diseases (IBD).
(2) Chronic inflammation associated with metabolic disorders, including obesity, type 2 diabetes (T2D) and cardiovascular diseases.
(3) Chronic bacterial, viral and parasitic infection, in which persistent immune activation sustains prolonged non-resolving inflammation.

All three categories share circadian rhythmicity as a common pattern, although with different manifestations.

### The circadian regulation of immune-mediated inflammatory diseases

**Fig. 2.**
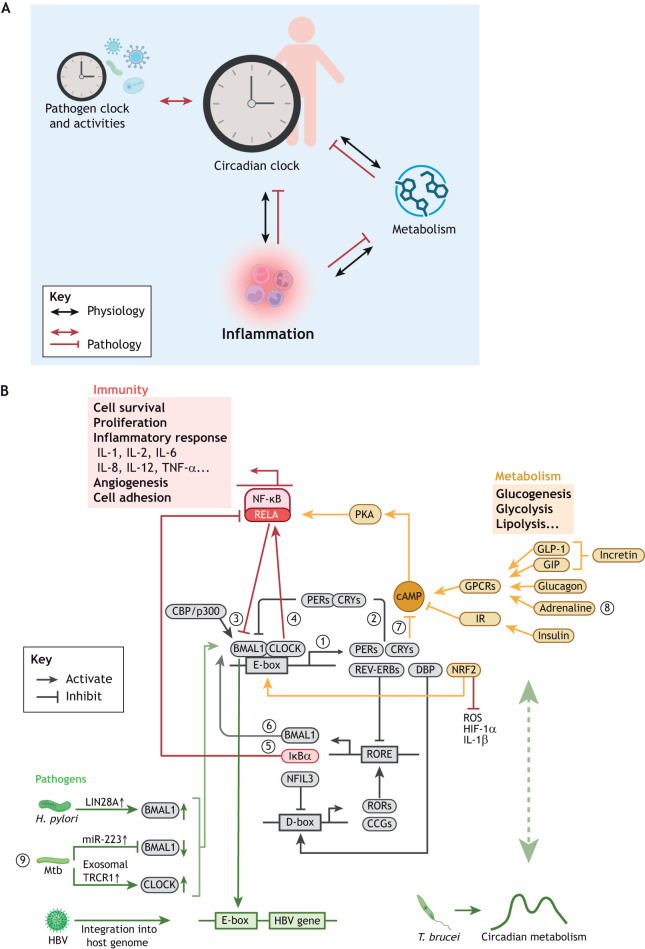
**Mutual regulation between the circadian clock and chronic inflammatory diseases.** (A) Conceptual illustration showing the interactions between the host circadian clock, inflammation, metabolism and pathogens. In physiological states (black arrows), the circadian clock time-gates inflammation and supports appropriately timed metabolic processes. Immune and metabolic processes also directly regulate each other. In chronic inflammatory diseases (red arrows), inflammatory cytokines and imbalanced metabolites persistently disrupt the circadian clock oscillations, resulting in a pro-inflammatory shift of homeostasis that hinders resolution. Additionally, in chronic inflammation caused by persistent infections, pathogens align their cycling activities with the host circadian clock to optimise survival. (B) Schematic showing the molecular mechanisms that link the core circadian machinery to host immune and metabolic processes, and how pathogens – with or without intrinsic clock – interact with the host circadian clock. Circadian, immune, metabolic and pathogen-related processes are coloured in grey, red, yellow and green, respectively. Key processes are numbered. ① and ② Core transcription-translation feedback loop. ③ RELA represses E-box-reporter activity driven by BMAL1−CLOCK in a way that is mechanistically synonymous to that of CRY1 repression. ④ CLOCK positively regulates NF-κB-mediated transcription via direct interaction with RELA. BMAL1 counteracts this activation effect by competing with RELA in binding to CLOCK. ⑤ RORα induces *IκBα* gene expression, which restrains NF-κB signalling. ⑥ RORs directly activate *Bmal1* gene expression. ⑦ CRY inhibits cAMP production. As cAMP activates PKA which subsequently activates NF-κB, CRY overall inhibits NF-κB activation. ⑧ Metabolic signals regulate cAMP production. ⑨ Pathogens interact with the host clock by regulating host clock component levels, integrating into the host genome, and potentially interacting with the host metabolism. CBP, CREB-binding protein; GIP, glucose-dependent insulinotropic polypeptide; GLP-1, glucagon-like peptide-1; GPCR, G protein-coupled receptors; HBV, hepatitis B virus; *H. pylori*, *Helicobacter pylori*; incretin, primarily includes gut peptides GLP-1 and GIP; IR, insulin receptor; Mtb, *Mycobacterium tuberculosis*; *T. brucei*: *Trypanosoma brucei*. Created in BioRender by Ray, D., 2026. https://BioRender.com/u9ka90d. This figure was sublicensed under CC-BY 4.0 terms.

Within this context, the circadian regulation of inflammation in immune-mediated inflammatory diseases (IMIDs) is relatively straightforward and clinically apparent, as inflammation is the direct consequence of immune disorders (Fig. 2A). While other systems can also be affected, they are mainly considered consequence of inflammation. The examples discussed below represent IMIDs of different organs and pathogenesis.

### Rheumatoid arthritis

RA symptoms follow a clear circadian pattern; most typically, joint stiffness and swelling of patients are most severe in the morning (Cutolo et al., 2008). This circadian pattern is associated with disrupted melatonin and cortisol rhythms, and the subsequently overactivated inflammatory responses. Melatonin stimulates pro-inflammatory cytokine production and enhances inflammation in affected joints. In healthy people, melatonin secretion increases from the evening and peaks at around 02:00, whereas in RA patients, the peak occurs earlier at midnight and persists longer (Sulli et al., 2002). This delayed and prolonged melatonin peak drives the accumulation of human pro-inflammatory cytokines overnight, including TNF-α (an early mediator of acute-phase inflammation), IFN-γ (an activator of macrophages and cell-mediated immunity), IL-1 (a potent immune-amplifying cytokine) and IL-12 (which is produced by antigen-presenting cells and activates adaptive immunity) (Petrovsky and Harrison, 1998; Cutolo et al., 2008). Cortisol, on the contrary, is a potent anti-inflammatory hormone. Although RA patients show similar cortisol rhythms as healthy controls, their cortisol levels are insufficient to fully restrain the nocturnal cytokine increase, especially of plasma IL-6, which shows a markedly higher morning peak (Crofford et al., 1997). Cortisol secretion depends on the HPA axis, which can be blunted by chronic inflammation in RA patients and further impair its anti-inflammatory ability (Cutolo et al., 2002). As a result, pro-inflammatory cytokines peaking in the early morning contribute to the symptoms of morning stiffness (Cutolo et al., 2008). During the day, as cortisol levels and sympathetic nervous system activity increase, cytokine production is inhibited and symptoms, such as stiffness and pain, are typically relieved compared to the morning. Persistent inflammatory activity also contributes to comorbidities in RA patients, including cardiovascular risks and osteoporosis (Szekanecz et al., 2019).

On the patient level, consistent with the earlier melatonin peak, RA patients show earlier chronotypes compared to the general population (Habers et al., 2021). Circadian misalignment may be important for RA development, as shift work is associated with a higher risk of RA (Butler et al., 2023).

Animal models have provided important insights into the mechanisms of RA circadian regulation. The collagen-induced arthritis (CIA) mouse model closely mimics many key pathological and genetic features of human RA (Holmdahl et al., 1989; Williams, 2004). In CIA mice, paw swelling (one of the standard measures of disease severity in the CIA model) in severely inflamed limbs and the levels of circulating proinflammatory cytokines are significantly higher during rest phase than active phase, mirroring daily symptom patterns in humans (Hand et al., 2016). Loss of *Bmal1*, *Cry1* and *Cry2* in CIA mice shifts fibroblast-like synoviocytes (i.e. mesenchymal-stem cell-derived cells that contribute to synovial fluid and articular cartilage production) towards a pro-inflammatory state and increases disease severity (Hand et al., 2016, 2019). Environmental circadian clock disruption, such as constant light, also interferes with peripheral clock gene oscillations in CIA mice and abolishes the daily variation of paw swelling, resulting in persistently elevated local inflammation (Hand et al., 2016).

### Asthma and other chronic lung diseases

Asthma is a chronic inflammatory airway disease affecting over 260 million people globally (Wang et al., 2023; Zhang et al., 2025) and shows strong circadian variation. Symptoms such as wheeze, cough and shortness of breath typically exacerbate overnight and peak in the early morning (Turner-Warwick, 1988; Skloot, 2002). These symptoms largely arise from inflammatory narrowing of the airway, driven by infiltration and activation of multiple immune cell types, including T helper 2 cells, dendritic cells, eosinophils, mast cells, innate lymphoid cells and neutrophils (Hammad and Lambrecht, 2021), many of which show circadian activities (Wang et al., 2022). Nocturnal symptoms affect 44-74% patients with asthma, varying by age and living environments, frequently causing sleep disruption and potentially impairing the daytime performance of patients (Pinyochotiwong et al., 2021; Turner-Warwick, 1988).

The circadian system contributes to the nocturnal exacerbations seen in asthma (Scheer et al., 2021). Systemically, the overnight decrease in adrenergic sympathetic tone and the increase in cholinergic parasympathetic activity promote bronchoconstriction, an asthma manifestation (Morrison et al., 1988; Soutar et al., 1977). The nocturnal decline of serum epinephrine seen in people may reduce inhibition of histamine release from pulmonary mast cells (Barnes et al., 1980), while melatonin promotes secretion of pro-inflammatory cytokines (including IL-1, IL-6 and TNF-α) from peripheral blood mononuclear cells (Sutherland et al., 2002). At the organ and cellular level, the lung ranks among the most rhythmic organs (Zhang et al., 2014). Circadian rhythmic genes in the lung are associated with asthma and other inflammatory lung disorders, including COPD, fibrosis and pneumonia (Sukumaran et al., 2011).

Evidence from both animal models and human studies further reveal involvement of the core circadian clock in a broad range of asthma pathogenic processes. Mice lacking *Bmal1* in myeloid cells developed markedly amplified ovalbumin-induced allergic asthma features, including increased eosinophils and IL-5 (a pro-inflammatory eosinophil-activating cytokine) (Zasłona et al., 2017). Surprisingly, reduced eosinophilic inflammation was observed in a house dust mite (HDM)-induced chronic lung allergy model (Hong et al., 2024). *Bmal1*-deficient mouse macrophages showed higher CCL2 (recruits inflammatory monocytes) and CXCL10 (recruits eosinophils, promotes airway inflammation) expression induced by LPS, and higher mannose receptor (linked to asthma and airway hyper responsiveness in humans and mice) expression induced by IL-4 (Zasłona et al., 2017). BMAL1 also binds to p53 and promotes its degradation, contributing to autophagy and PM2.5-aggravated asthma in mice (Chen et al., 2023). In an intra-nasal Sendai-virus-infection asthma mouse model, global *Bmal1* deletion worsened bronchiolitis and raised resting airway resistance (Ehlers et al., 2018). *Rev-erbα* also gates daily airway hyper-responsiveness, as its loss abolishes the daily variation in asthma severity in mice (Durrington et al., 2020). In humans, reduced airway *Bmal1* expression was consistently observed in both adults with asthma and infants with respiratory syncytial virus bronchiolitis compared to time-matched healthy controls in their own cohorts (Ehlers et al., 2018). Primary airway epithelial cells from children with allergic asthma maintain rhythmic expression of core circadian clock genes, although *Per3* and *Rev-erbα* showed increased amplitudes of circadian oscillation. Furthermore, downstream genes whose rhythmicity of expression was changed were enriched in cytokine-related pathways (Powell et al., 2024). Sex differences regarding asthma are also evident, as both acute and chronic HDM exposure revealed stronger circadian variations in inflammatory response in female mice rather than in male mice (Srinivasan et al., 2023a,b). Similarly, in humans, a large UK Biobank study found that females working night-shifts have a higher risk of asthma than females working during the day, an association not observed in males (Maidstone et al., 2025).

A range of other chronic lung diseases with inflammatory components also exhibit circadian regulation, as reviewed by Giri and colleagues (2022). In chronic obstructive pulmonary disease (COPD), symptoms and disease severity show daily variation and are often worse early morning or late night, while night-time symptoms are more common in the chronic bronchitis subtype than that of emphysema (Tsai et al., 2007; Scichilone et al., 2019). Loss of REV-ERBα in mice exaggerates cigarette-smoke-induced inflammatory responses (Wang et al., 2021), while inhibition of RORγ (officially known as RORC) suppresses release of IL-17A (a pro-inflammatory cytokine) in COPD patients (Desai et al., 2022).

Human idiopathic pulmonary fibrosis (IPF) risk is associated with circadian disruption, such as shift work and evening chronotype (Cunningham et al., 2020). Mouse pulmonary fibrosis (PF) models show a gain of amplitude and lack of synchrony within fibrotic tissue, driven by infiltration of mesenchymal cells, an important contributor to PF pathogenesis (Cunningham et al., 2020). Mechanistically, BMAL1 is required for TGF-β1-induced fibroblast-to-myofibroblast differentiation and epithelial-to-mesenchymal transition in human lung cell lines, both contributing to fibrosis (Dong et al., 2016). REV-ERBα inhibits myofibroblast activation through the transcription factor TBPL1 in bleomycin-induced fibrosis in mice, and consistently, increases in human IPF (Cunningham et al., 2020).

### Inflammatory bowel disease

Inflammatory bowel disease (IBD) is a group of chronic relapsing intestinal inflammatory conditions, including Crohn's disease and ulcerative colitis (Zhang and Li, 2014). Most patients with IBD suffer from poor sleep quality. It is particularly prevalent in those with active disease (78.1%) compared with those in clinical remission (64.9%) (Marinelli et al., 2020). In turn, sleep disturbance and circadian disruption are associated with exacerbated inflammation and worse disease outcomes (Swanson et al., 2022). Swanson and colleagues describe increased serum TNF levels in IBD patients with sleep disturbance, which indicates increased systemic inflammation, although sampling time was not specified (Swanson et al., 2022). In mice with colitis induced by *IL-10*-knockout, rhythmicity of core clock genes in colonic tissues is blunted; *Tnf* and *Ifng* expression is prominently elevated, peaking around the late rest phase (Niu et al., 2024).

### The circadian regulation of inflammation in chronic metabolic disorders

Many chronic metabolic disorders, most notably obesity, type 2 diabetes (T2D) and cardiovascular diseases, are characterised by persistent low-grade inflammation (Yadav et al., 2024). In addition to the circadian-immune interactions discussed above, circadian regulation in these conditions involves an additional metabolic layer (Fig. 2A). The close evolutionary and mechanistic links between metabolism and immunity provide a conceptual foundation for this discussion (Box 2), and their detailed molecular crosstalk has been extensively reviewed (Reddy et al., 2019; Lee and Olefsky, 2021; Saltiel and Olefsky, 2017; O'Neill et al., 2016). In this Review, we extend the framework and focus on the circadian clock, a higher-order regulatory system that coordinates both domains and examine how circadian regulation shapes immune-metabolic interactions in chronic metabolic diseases.

Box 2. Why are metabolic and immune disorders so closely connected? Metabolic and immune systems together support survival by enabling organisms to withstand starvation and defend against infection and injury. Since both processes are energy-intensive to maintain, their integration allows optimal energy allocation, which is essential in resource-limited environments (Hotamisligil, 2006). Evidence across species at different evolutionary levels support this link. In *Drosophila melanogaster*, the fat body is a multifunctional organ that integrates nutrient storage, endocrine signalling and immune responses (Huang and Perrimon, 2026). In mammals, although these functions are distributed across the liver, adipose tissue and immune system (Huang and Perrimon, 2026), immune-metabolic integration remains fundamental at both intra- and inter-cellular levels. Immune cells dynamically adjust glycolysis, oxidative phosphorylation and lipid handling to support their functional state (O'Neill et al., 2016). For example, Kupffer cells can internalise apolipoprotein-B-containing particles and adopt an inflammatory transcriptional profile that contributed to the progression toward steatosis, as shown in a mouse model of dyslipidaemia (Di Nunzio et al., 2024). Intercellular coordination is also evident. During fasting, glucocorticoid receptor signalling in hepatic macrophages suppresses TNF secretion and, thereby, promotes hepatocyte GR/PPARα-driven activation of ketogenesis (Loft et al., 2022; Stephan and Zucker, 1972). The close spatial organization of Kupffer cells and hepatocytes further facilitates their bidirectional metabolic and immune crosstalk (Fantini and Norata, 2025).

While these highly integrated nutrition- and pathogen-sensing mechanisms have been evolutionarily advantageous, they have not yet adapted to the lifestyle of modern society, where nutrition excess becomes a normality, and obesity and diabetes are increasingly prevalent (Bhupathiraju and Hu, 2016; Fagiani et al., 2022). The mutual causal relationship between metabolic diseases and chronic inflammation has been well-established since the discovery of TNF-α expression in obese mice (Hotamisligil et al., 1993). Mechanistically, chronic nutritional surplus, such as obesity, is a proinflammatory state characterised by oxidative stress, which increases macrophage infiltration and activation in adipose tissue (Furukawa et al., 2004). Macrophage- and adipocyte-derived cytokines, in turn, promote insulin resistance and inhibit the anti-inflammatory effect of insulin, thereby, further enhancing the metabolic disorder and worsening the vicious cycle (Dandona et al., 2004; Hotamisligil et al., 1993).

An early and fundamental question in immunometabolism, articulated by Hotamisligil in 2006, concerns how inflammatory reactions are restrained during physiological nutrient fluctuations and how nutrients are cleared in their respective tissues (Hotamisligil, 2006). With advances in circadian biology, it is now increasingly evident that the circadian system may be the regulatory mechanism that the question sought. Several key features make the circadian clock an ideal coordinator of immune-metabolic homeostasis. First, the core function of the clock is to anticipate predictable daily challenges, including fluctuations in physiological nutrient and energy levels, aligned with activity and rest cycles. Second, the clock temporally orchestrates physiological processes to occur at the most appropriate times and in optimal sequence. It temporally tunes the amplitude and duration of immune activity, ensuring that inflammatory responses remain transient and energetically compatible with concurrent metabolic demands of the active phase. Third, tissue-specific circadian regulations introduce a spatial element to biology. For example, in mice, the expression peaks of core clock components generally occur earlier in the SCN compared to the liver; whole-cell rhythmic proteins peak in the SCN at circadian times 2 or 14 (CT2 and CT14, respectively), and between CT16 and CT18 in the liver (Otobe et al., 2026) – CT being the 24-hour representation of the 24-h phase in the endogenous circadian cycle, with CT0 indicating the start of the subjective day and CT12 the start of the subjective night. Growing physiological evidence in humans and mice supporting this rationale (reviewed by Carroll et al., 2019; Panda, 2016; Guan and Lazar, 2021), highlight the key role of circadian regulation in immunometabolism settings.

Obesity and T2D are characterised by markedly dampened or altered circadian rhythms in metabolic and immune processes. The ‘dawn phenomenon’ is a well-known abnormally high increase in early-morning blood glucose observed in patients with diabetes (Peng et al., 2022). It represents an exaggerated version of the physiological circadian increase in blood glucose levels seen in healthy individuals. In diabetes, this effect becomes more pronounced because of impaired glucose tolerance and reduced insulin sensitivity (Morris et al., 2015; Barua et al., 2024). In healthy individuals, adipose tissue also exhibits circadian activity in glucose and lipid metabolism, and adipocytokine secretion (Zinna et al., 2025). However, these natural oscillations are dampened in obese and diabetic conditions, as the rhythmic expression of core clock genes is progressively disrupted with disease progression (Rivera-Alvarez et al., 2025; Ando et al., 2005).

Beyond metabolic tissues, the circadian clock also exerts a profound influence on cardiovascular physiology, generating daily variations in key parameters, such as blood pressure (Millar-Craig et al., 1978) and heart rate variability (Bonnemeier et al., 2003). Disruption of such temporal coordination – whether arising from obesity or related conditions, such as hypertension, hyperglycaemia, T2D or dyslipidaemia, or from irregular lifestyle patterns – contributes to increased cardiovascular risks (Powell-Wiley et al., 2021; Lecacheur et al., 2024).

### Circadian regulation in chronic infection

In addition to the host circadian network, chronic infection involves host-pathogen interactions, introducing further temporal complexity (Fig. 2). As aptly phrased by Diallo et al., the question becomes “for whom the clock ticks”: circadian regulation of host physiology and behaviour influences pathogen survival inside the host. At the same time, pathogens exploit or disrupt temporal organisation of the host for their own fitness. Additionally, some parasites possess intrinsic clocks that align with or manipulate host rhythms (Diallo et al., 2020). Among chronic bacterial infections, we will discuss *Helicobacter pylori* (*H. pylori*) and *Mycobacterium tuberculosis* (*M. tuberculosis*) as examples from different organs and clinical aspects.

*H. pylori* is a carcinogen for gastric cancer (Navashenaq et al., 2022). The common manifestation of its infection, i.e. peptic ulcers, frequently causes nocturnal pain from midnight till early dawn (Saitohl et al., 2000). *H. pylori* infection has been mechanistically linked to direct circadian disruption, as this bacterium has been shown to induce *Bmal1* expression through transcriptional activation of *LIN28A* (encoding an RNA-binding protein acting as an translation reprogramming factor), which promotes inflammatory responses, such as TNF-α secretion, and contributes to persistent tissue injury (Li et al., 2019).

In adults with pulmonary tuberculosis (TB), coughing – a predictor for transmission risk and treatment outcome – peaks during the day (Proaño et al., 2017). *M. tuberculosis* infection inhibits *Bmal1* expression in mouse peritoneal macrophages and activates matrix metalloproteinases (a family of enzymes that degrade extracellular matrix), potentially through interaction with BMAL1-miR-223 (an immune-regulating microRNA in myeloid cells) (Lou et al., 2017). REV-ERBα in the human THP-1 cell line enhances *M. tuberculosis* clearance by repressing *IL-10* expression (Chandra et al., 2013) and promoting autophagy (Chandra et al., 2015). Although most mechanistic studies linking *M. tuberculosis* infection to the clock machinery were limited to *ex vivo* cells, a more recent study revealed that TRCR1, a long non-coding RNA targeting the CLOCK protein, induces antimicrobial immunity against tuberculosis and improves the efficiency of the Bacillus Calmette–Guérin vaccine that protects against TB (Yu et al., 2026). Morning administration of this vaccine in healthy adult volunteers induces stronger trained immunity responses than evening dosing, further highlighting the importance of circadian timing in host defence against *M. tuberculosis* (de Bree et al., 2020).

*H. pylori* and *M. tuberculosis* infection illustrate how circadian physiology shapes chronic inflammatory responses differently in the stomach and lung, regarding their manifestations and immunopathology. Major symptoms of *H. pylori* infection mostly exhibit in the gastrointestinal system, for example bloating, nausea and altered acid production (Öztekin et al., 2021). The clear circadian rhythm of intragastric acidity displayed in healthy males shows significantly reduced amplitude in *H. pylori*-positive patients (Saitohl et al., 2000). By contrast, symptoms of TB are more varied and systemic, including chronic cough, fever and night sweats (Guzmán-Téllez et al., 2024). Innate immune responses are key drivers of lung pathology (Liu et al., 2017a), with macrophages being the major host cell type in *M. tuberculosis* infection (Russell et al., 2025). *H*. *pylori*, on the other hand, induces both innate and adaptive immune responses (Wilson and Crabtree, 2007), therefore involving more-complex circadian−immune crosstalk, as discussed above.

Compared with bacterial infections, chronic viral infections are interesting because the pathogen is even more dependent on the host, since viruses lack intrinsic circadian machinery. Here, we use Hepatitis B virus (HBV) as an example for its significant global impact on human health, and for its representativeness of such a host-dependent mechanism, as the liver is among the most rhythmic organs (Skrlec and Talapko, 2022). HBV integrates into the hepatocyte genome, creating a persistent source of hepatitis-B-surface antigen (HBsAg) expression throughout chronic infection and might drive dysfunctional T-cell responses (Iannacone and Guidotti, 2022). Meanwhile, because integrated viral genome relies on the host transcriptional machinery for gene expression, this also places the viral expression under direct host circadian transcriptional control. For example, the E-box motif (a DNA sequence that interacts with bHLH proteins), which is conserved among HBVs and higher primates, enables BMAL1 binding to HBV genome sequences and enhances viral promoter activity (Zhuang et al., 2021). Moreover, REV-ERBα regulates HBV entry into hepatocytes by directly regulating the protein expression of the transmembrane transporter NTCP (officially known as SLC10A1), a functional receptor for HBV (Zhuang et al., 2021). Clinically, a higher failure rate of HBV vaccination was reported in shift workers (Kim and Chae, 2024), indicating the potential involvement of circadian regulation in anti-HBV responses.

Many chronic parasitic infections also exhibit circadian variation in their symptoms and disease progression. As in bacterial and viral infections, the circadian system of the host plays a key role in shaping these dynamics. For instance, in mice, the severity of chronic cutaneous leishmaniasis varies by time of infection and is at its lowest when *Leishmania* infection happens at the beginning of the rest period. Such rhythmicity is regulated by the host-cell-intrinsic circadian clock, as it is abolished in neutrophils and macrophages of mice that lack *Bmal1* (Kiessling et al., 2017). What distinguishes parasitic infections from the previous two categories, however, is that some parasites possess their own endogenous circadian mechanisms, enabling them to autonomously regulate their physiology. For example, *Trypanosoma brucei* (a parasite causing African trypanosomiasis in humans) was found to possess an intrinsic circadian clock that generates rhythmic expression of ∼10% of its genes *in vitro* in a post-transcriptional manner (Rijo-Ferreira et al., 2017). Remarkably, some parasites can also align their cyclic activities with those of their hosts, exploiting host circadian processes to optimise their fitness. Malaria serves as an important example here, as the cell cycles of *Plasmodium* follow multiples of approximately 24 h, and disruption of this synchrony increases the burden for both replication and transmission (O'Donnell et al., 2011). Together, these examples illustrate that circadian regulation is a pervasive and evolutionarily conserved dimension of host−pathogen interactions.

## Effects of chronic inflammatory diseases on the circadian clock and health of the host

In the previous sections, we examined how the circadian clock shapes inflammation and host-pathogen interactions. In this section, we shift the perspective to the reverse direction, i.e. how chronic inflammatory diseases perturb the circadian system (Fig. 2A, red arrows). Although chronic inflammatory diseases encompass a wide spectrum of conditions, their diverse manifestations often converge on a few common pathways that feed back onto the circadian machinery. Therefore, we will focus on the shared mechanisms through which chronically altered inflammatory and metabolic homeostasis affect circadian regulation, and how such perturbations of the central temporal node may drive multimorbidity.

It is well established that chronic inflammation and nutritional challenge can reshape the circadian clock. Chronic inflammatory arthritis in mice broadly alters circadian transcriptional programmes and energy metabolism, both within the joints and in distal metabolic tissues, such as skeletal muscle and liver (Downton et al., 2022). In inflammatory airway conditions, TGF-β – a key cytokine driving lung fibrosis – upregulates *Bmal1* and *Npas2*, and downregulates *Pers*, *Rev-erbα*, *RORα* and *Dbp* in mouse lungs (Dong et al., 2016). Exposure to cigarette smoke also suppresses *Rev-erbα* in mouse lungs (Vasu et al., 2009). High-fat diet in mice reduces the oscillation amplitude of peripheral clock genes in adipose tissue and the liver, with a stronger dampening effect on the oscillation of core circadian clock gene expression in the adipose tissue (Kohsaka et al., 2007). A high-fat diet also profoundly reorganises circadian transcriptome and metabolome in the liver of mice by impairing CLOCK−BMAL1 chromatin recruitment, through a fatty-acid-sensing mechanism (Eckel-Mahan et al., 2013). In humans, obesity is associated with increased clock gene expression in peripheral blood mononuclear cells (Tahira et al., 2011). Infection-induced inflammation dampens the amplitude of host circadian oscillation; for example, *Salmonella* downregulates *Per2* expression in mice; parasite infection, such as *T. brucei*, advances the circadian phase of activity and *Per2* gene expression in peripheral organs of host mice (Rijo-Ferreira et al., 2018).

The perturbation of chronic inflammation and metabolic stress to the circadian clock can happen through a network of cytokines, hormones and transcription factors (Cermakian et al., 2014). Cytokines, such as TNF-α, inhibit the transactivation effect of CLOCK−BMAL1 on E-box driven transcription (Cavadini et al., 2007), while IFN-α directly decreases CLOCK and BMAL1 protein levels, thereby suppressing the core clock oscillation (Koyanagi and Ohdo, 2002). Inflammation-induced upregulation of leptin, prostaglandin E_2_ and glucocorticoids also affects clock gene expression, and causes a phase shift (a change in the timing of expression peaks of clock components) in mice and humans (reviewed previously by Cermakian et al., 2014).

Importantly, many of these diverse signals converge on shared transcriptional regulators, most notably NF-κB, which engages in bidirectional molecular coupling with the core clock machinery. For example, in mouse cartilage explants, IL-1β exposure dampens the circadian expression rhythms of *Per2* and *Cry1* (components in the negative arm of the clock) in an NF-κB-dependent manner, the effect being rescued by inhibiting IκB kinase, an activator of NF-κB (Guo et al., 2015). Glucocorticoids, whose levels are strongly circadian, rhythmically suppress NF-κB action via the glucocorticoid receptor (Webster et al., 2002).

Mechanistically, inflammatory transcription and circadian timing are bi-directionally coupled through direct interactions between RELA (a subunit of NF-κB essential for its activation and nuclear translocation), CLOCK−BMAL1 and their shared co-regulatory surfaces (Fig. 2B). RELA competes with the homologous BMAL1 coactivators CBP and p300 (officially known as CREBBP and EP300, respectively) in binding the transactivation domain (TAD) of BMAL1, thereby repressing the transcriptional activity of the BMAL1−CLOCK complex (Shen et al., 2021). This repression happens in a way parallel to that of CRYs, which normally represses the CLOCK−BMAL1 complex through the same BMAL1 TAD docking site (Narasimamurthy et al., 2012). Consequently, expression of the genes downstream BMAL1−CLOCK is inhibited by NF-κB. For example, in mice, LPS-induced activation of NF-κB inhibits gene expression of members of the *Per*, *Crys* and *Rev-erb* families. For example, in response to LPS (component of gram-negative bacteria)-induced acute inflammation model in mice (Hong et al., 2018). These findings provide a concrete route by which inflammatory NF-κB activation dampens the transcriptional amplitude of the core clock. Conversely, clock components can shape inflammatory transcription. CLOCK acts as an activator of NF-κB-mediated transcriptional programmes by directly interacting with RELA (Spengler et al., 2012). RORα (officially known as RORA) supports oscillator robustness by promoting *Bmal1* transcription via ROREs (Akashi and Takumi, 2005) and also provides an anti-inflammatory counterweight by inducing expression of IκBα, the major inhibitor of NF-κB signalling (Delerive et al., 2001). Further downstream, NF-κB itself is a transcription factor, and its target genes are broadly involved in inflammation processes and cell survival (Liu et al., 2017b).

Functionally, NF-κB and BMAL1 are both critical in regulating circadian and inflammation processes. In mice, basal NF-κB activity is required for maintenance of normal circadian rhythm behaviour (Hong et al., 2018), and NF-κB constitutive activation in neural tissues affects the locomotor activity by extending the circadian period length in constant darkness and slowing down their re-entrainment to an advanced light phase (Shen et al., 2021). BMAL1 has a notable protective role against neuroinflammation. The BMAL1–CLOCK or BMAL1–NPAS2 heterodimer protects mice from neurodegeneration and neuronal oxidative stress and is essential for resting-state functional connectivity (Musiek et al., 2013). Astrocyte-specific *Bmal1* loss triggers a cell-autonomous reactive inflammatory program linked to impaired glutathione-related signalling (Lananna et al., 2018). Together, these findings support an integrated mechanistic network in which NF-κB−CLOCK competition for BMAL1 binding, CLOCK-mediated activation of NF-κB output and ROR-dependent NF-κB inhibition jointly couple chronic inflammation with circadian dysfunction, in which RELA serves as a key node (Fig. 2B).

The above network further interfaces with metabolic processes through the cyclic AMP (cAMP)−NF-κB pathway. cAMP is a second messenger synthesised upon GPCR activation and activates protein kinase A (PKA), which then activates NF-κB by phosphorylating RELA (Narasimamurthy et al., 2012). CRY1 limits cAMP production by binding to adenylyl cyclase and, subsequently, inhibits activity of NF-κB (Narasimamurthy et al., 2012). Metabolic-related neuro and endocrine signals also regulate cAMP production, such as fasting-state glucagon (Jiang et al., 2001; Zhao et al., 2023), fed-state insulin (Zhang et al., 2005), incretin hormones, such as GLP-1 (a cleaved product of GCG) and GIP (Zheng et al., 2024; Mayendraraj et al., 2022), and β-adrenergic signalling (De Jong et al., 2023). These findings suggest a potentially bidirectional molecular interaction between the circadian clock and metabolism, where CRY−cAMP−NF-κB is a key connection, although its functional consequence in human metabolic disease contexts still needs further investigation (Fig. 2B).

Oxidative stress represents another major intersection between inflammation and circadian disruption. Reactive oxygen species (ROS) are produced rhythmically as by-products of the mitochondrial metabolism and, normally, are tightly regulated by circadian antioxidant defences (Patel et al., 2014). In this process, NRF2 is an important circadian clock-regulated transcription factor that inhibits ROS, and in macrophages, it inhibits its downstream pro-inflammatory cytokines IL-1β and IL-6 (Early et al., 2018; Kobayashi et al., 2016). NRF2 is also essential for maintaining a normal circadian pace in mouse hepatocytes (Wible et al., 2018). Mechanistically, NRF2 binds to the promoter sites of *Cry2* (Wible et al., 2018) and *Rev-erbα* (Yang et al., 2014), promoting their expression, thereby indirectly inhibiting the BMAL1−CLOCK transcriptional activity (Wible et al., 2018). Conversely, the BMAL1−CLOCK complex drives the daily oscillating NRF2 expression and NRF2–glutathione-mediated antioxidant defence pathway activity in the mouse lung, gating susceptibility to oxidative injury and fibrotic remodelling (Pekovic-Vaughan et al., 2014). When the antioxidative defences are overwhelmed, such as by chronic inflammation or metabolic overload, excessive ROS causes oxidative stress that feeds back on the circadian clock. At such states, ROSs inhibit the activity of sirtuin 1 (SIRT1), an NAD^+^-dependent deacetylase that modulates BMAL1 acetylation (Nakahata et al., 2008). Loss of SIRT1 activity relieves the deacetylation-mediated inhibition of the RELA subunit of NF-κB (Yeung et al., 2004), further amplifying inflammatory gene expression. Together, NRF2 is a key molecule linking reciprocal crosstalk between the circadian clock and redox signalling (Fig. 2B).

Collectively, in the network of metabolism, circadian rhythms and the immune system, diverse signals converge upon a few central transcriptional nodes that interface with the circadian clock, further disseminating altered temporal cues across multiple physiological systems by forming a ‘bottleneck-like’ signalling network.

## Current therapies and treatments

### Limitations of conventional therapeutics and advantages of circadian approaches

**Fig. 3.**
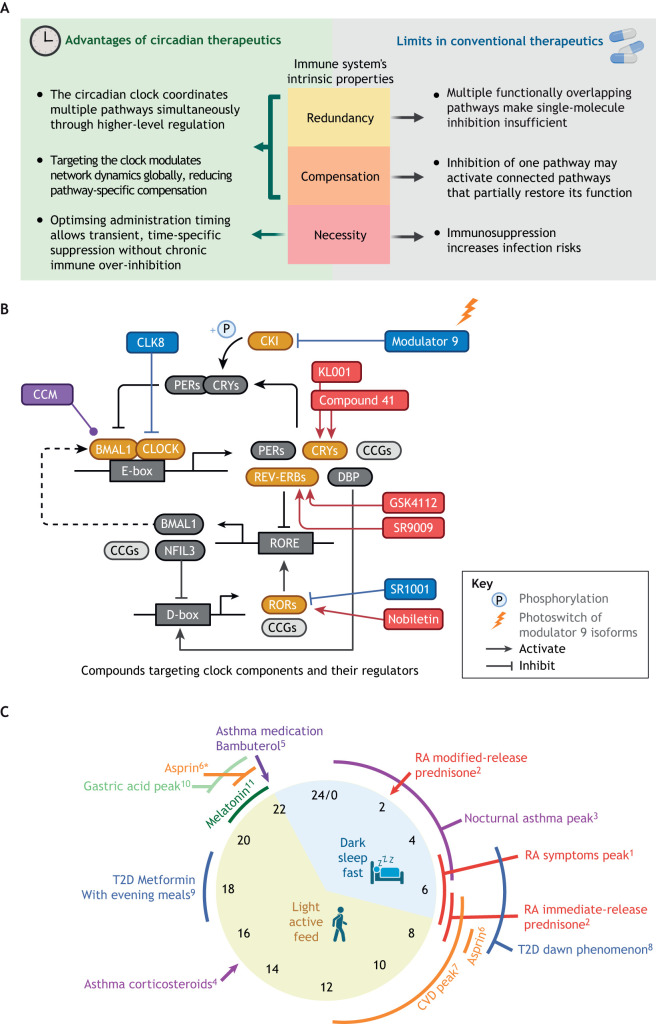
**Advantages and approaches of circadian therapies.** (A) Circadian therapies may help overcome the bottlenecks faced by conventional anti-inflammatory medications, which arise from the intrinsic properties of the immune system. (B) Compounds targeting clock components and their regulators. Indicated in red are activators, agonists or stabilizers. Indicated in blue are inhibitors or inverse agonists. Indicated in purple are modulators that do not belong to the above classifications Indicated in yellow are clock components and their regulators that are pharmacologically targeted. Indicated in grey are other clock components. CCM, core circadian modulator; CKI, casein kinase 1; CLK8, CLOCK inhibitor 8. (C) Circadian phases of chronic inflammatory disease symptoms showing the optimised timing of medicine administration. Coloured text highlights connections to rheumatoid arthritis (RA, red), asthma (purple), cardiovascular disease (CVD, orange), type 2 diabetes (T2D, blue), *H. pylori* infection (light green) or melatonin (dark green). Superscript numbers 1-11 indicate references ^1^Cutolo et al., 2008, ^2^Buttgereit et al., 2008; ^3^Li and Reed, 1985, ^4^Beam et al., 1992, ^5^D'Alonzo et al., 1995; ^6^Cooke-Ariel et al., 2015, ^7^Buurma et al., 2019; ^8^Peng et al., 2022, ^9^Bailey, 2024; ^10^Saitohl et al., 2000; ^11^Xie et al., 2017. The asterisk (*) indicates that evening intake of Asprin may be optimal for individuals not exhibiting a J-curve blood pressure, often including patients with diabetes, congestive heart failure or chronic kidney disease (Cooke-Ariel et al., 2015). CLK8: CLOCK inhibitor 8. Created in BioRender by Ray, D., 2026. https://BioRender.com/0xa7d37. This figure was sublicensed under CC-BY 4.0 terms.

Treating chronic inflammatory diseases has long been challenging due to the fundamental characteristics of the immune system itself. As highlighted by Tabas and Glass, the three properties of inflammation that make it critical for survival – i.e. redundancy, compensation and necessity – also make it resistant to therapeutic modulations (Tabas and Glass, 2013). Redundancy and compensation of the inflammatory network provide robustness and resilience against perturbations to a single component. However, they also limit the effectiveness of therapies that target single pathways because inhibition of one pathway can be compensated for by activation of pathways performing a similar function. Even if multiple pathways can be successfully targeted, the necessity of inflammation for host defence means that oversuppression of immune responses often carries unacceptable infection risks, especially on long timescales (Fig. 3A). As a result, although the disease-modifying agents have made remarkable progress in certain areas – such as anti-TNF-α antibodies in treating rheumatoid arthritis (Feldmann et al., 2010), and low-doses of methotrexate (Feldmann et al., 2010) and anti-IL-1β antibodies (Ridker et al., 2011) in treating inflammatory atherosclerosis  – treatment of chronic inflammatory diseases is still using more-conservative symptomatic treatments and are limited to a narrow risk/benefit window (Tabas and Glass, 2013).

Circadian biology is offering promising new solutions to these limitations. Just as the inherent properties of inflammation give rise to therapeutic challenges, the intrinsic regulatory design of the circadian clock offers a means to overcome them (Fig. 3). First, the circadian clock can overcome immune-system redundancy by serving as an apex regulator. Second, targeting the circadian clock, which orchestrates different aspects of cellular processes, has the potential to yield effects comparable to those combining several conventional drugs that target separate pathways. Importantly, circadian regulation does not require continuous or absolute inhibition of inflammation. Instead, it adjusts the timing and amplitude of immune activity without fully abolishing it. For instance, short-acting or clock-targeting drugs can be administered at the optimal circadian phase to transiently suppress inflammation or restore the physiological circadian pattern of inflammation regulation rather than overriding it, and is discussed in the following section.

### Circadian-based interventions

Circadian medicine seeks to optimise treatment efficacy by aligning therapeutic interventions with the internal timing of the body, and its approaches have been summarised into three major categories: (1) developing compounds that directly modulate clock function, (2) adjusting the administration schedules of existing drugs and (3) restoring circadian synchrony via behavioural or lifestyle interventions (Sulli et al., 2018). Some advances have been made with encouraging results reported in a range of chronic inflammatory conditions, including rheumatoid arthritis, asthma, osteoarthritis and cardiovascular disease (as reviewed by Jacob et al., 2020). Direct targeting of the clock has not been attempted, as this may exert pleiotropic effects. However, some encouraging developments suggest targeting circadian amplitude may offer benefits without shifting sleep−wake rhythms (Zhao et al., 2016). In this section, we build on these insights to further elucidate how circadian medicine offers novel opportunities in overcoming the limitations of conventional therapies.

### Clock-modulating compounds

Numerous small-molecule modulators targeting circadian clock components have shown therapeutic potential in metabolic and inflammatory disease models (Sulli et al., 2018; Jacob et al., 2020; Chen et al., 2018). Representative examples related to biological processes in chronic inflammatory diseases include CRY-stabilizers, such as KL001 that lengthens the circadian period and suppresses gluconeogenesis in primary mouse hepatocytes (Hirota et al., 2012). Compound 41 derived from KL001 was further shown to enhance glucose clearance in diet-induced obese mice (Humphries et al., 2016). A CLOCK ligand CLK8 disrupts the BMAL1−CLOCK interaction and inhibits CLOCK nuclear translocation, both in the human osteosarcoma (U2OS) cell line and in mouse livers (Doruk et al., 2020). For REV-ERBs, GSK4112 competes with heme in binding to REV-ERBα and represses glucogenesis in primary mouse hepatocytes (Grant et al., 2010). Another REV-ERBα agonist, SR9009, inhibits inflammation following myocardial infarction in mice and leads to significantly improved survival rate (Stujanna et al., 2017). The ROR inverse agonist SR1001 represses mouse T helper 17 (T_H_17) cell development and cytokine expression in human peripheral blood mononuclear cells (PBMCs), and suppresses the disease severity in a mouse multiple sclerosis model (Solt et al., 2011). In contrast to the synthetic ligands above, nobiletin, a citrus-derived natural flavonoid that binds and activates RORA, and enhances circadian amplitude (He et al., 2016), has exhibited broad metabolic protective effects against insulin resistance (Mulvihill et al., 2011) and disruptions to GLP-1 rhythms (Martchenko et al., 2022) in mouse models fed a high-fat-diet. Another reason to highlight ROR-targeting compounds is that many novel modulators in this class are being identified and tested in clinical trials to treat conditions, such as psoriasis, multiple sclerosis and dry eye syndrome (Ribeiro et al., 2021). Among the drugs being evaluated – with phase-I clinical trial results available – VTP-43742 demonstrated efficacy in patients with psoriasis within four weeks and without drug-related cardiac abnormalities (Gege, 2017). Although topically applied GSK2981278 ointment did not improve psoriasis in patients (Kang et al., 2018), its derivative has shown improved cytokine-inhibition effects in mice (Qi et al., 2024) (Fig. 3B).

Despite the broad range of clock-targeting compounds and their regulatory abilities in cell and animal models, concerns remain regarding their translation of application in humans. Components directly targeting clock proteins may have unexpected consequences due to the presence of clock machinery across the body and the global regulatory role of the circadian clock. Therefore, while phase-I clinical trials were efficient in filtering out compounds that have raised safety concerns (Ribeiro et al., 2021), the clock-targeting modulators require refinement to achieve better precision.

To achieve therapeutic benefits while minimising the undesired disruptions, efforts have been made in several directions: (1) control the circadian phase of drug administration and activation, (2) target components closely related to – but not part of – the clock machinery and, (3) altering the transcriptional activation effect of clock components rather than changing their expression levels and, finally, (4) identifying agents to increase circadian amplitude (Zhao et al., 2016).

Modulator 9, the photoswitchable inhibitor of casein kinase I (CKI) (Kolarski et al., 2021), represents a promising recent advance combining the above listed points (1) and (2). Unlike conventional CKI inhibitors that rely solely on pharmacokinetics to define their window of action, modulator 9 can be switched between isomeric states with light, showing higher selectivity towards CKIδ upon irradiation. This allows reversible inhibition of the circadian period and phase after a single administration, which has been validated in multi-day experiments by using the human U2OS cell line and mouse tissue explants. This strategy enables manipulation of circadian dynamics with high temporal precision and, potentially, spatial precision when combined with *in-situ* photoisomerization in response to visible light (Kolarski et al., 2021). The novel compound Core Circadian Modulator (CCM) – representing above listed point (3) – selectively targets BMAL1 by binding to its PAS-B domain; this alters BMAL1−CLOCK transcriptional activity without changing expression levels of BMAL1 (Pu et al., 2025), allowing for modulation of BMAL1 function as a transcriptional factor independently to direct protein-level effects of BMAL1. Functionally, the effect of CCM treatment on macrophage gene expression profiles is distinct compared with BMAL1 knockdown, indicating an effect beyond the traditional binary agonist/antagonist classification that provides another option for circadian modulation (Pu et al., 2025). These compounds represent novel avenues for the development of circadian clock modulators to manipulate rhythmic gene expression programmes while minimising the risk of globally disrupting the clock (Fig. 3B).

### Optimising timing of drug administration

For therapeutics that do not directly target the circadian machinery, optimising the timing of administration can markedly improve clinical outcomes by enhancing efficacy and reducing side effects without altering the drug itself, offering a cost-effective strategy that complements new drug development. The half-life of immediate-release drugs should be considered together with their dosing time, for the time window of a therapeutic concentration to align with the intrinsic circadian rhythms. Melatonin, as one of the best known examples to improve sleep disorders, has a short elimination half-life within an hour (Tordjman et al., 2017). Melatonin taken before sleep, therefore, enhances the natural nocturnal melatonin peak of the body to improve sleep latency (Xie et al., 2017). Another way to control dosing time is through modified release systems, which is especially suitable for conditions that peak during sleep. For example, in RA patients, modified-release prednisone administered at bedtime – enabling a delayed drug release in the early morning (at ∼02:00) that matches the timing of inflammatory cytokines increase – leads to a significantly reduced duration of morning joint stiffness and decreased IL-6 levels compared to standard immediate-release prednisone taken between 06:00-08:00 (Buttgereit et al., 2008). More examples are summarised in Fig. 3C. Mistimed dosing, by contrast, can compromise efficacy. For example, in patients with atherothrombotic myocardial infarction, platelet inhibition due to mid-morning intake of clopidogrel is notably weaker compared to intake at other times of the day (Kozinski et al., 2011). Insufficiently inhibited platelet aggregation in the morning, accompanied by a physiological morning peak of blood pressure, could contribute to higher risks of ischaemic cardiac events (Kozinski et al., 2011). Time-of-day-dependent treatment effects have also been extensively reported in other chronic diseases as reviewed previously (Dallmann et al., 2016; Jacob et al., 2020), underscoring the broader therapeutic potential of circadian timing-based strategies.

The clinical implementation of chronotherapy faces biological and practical challenges. Humans show large interindividual variations in chronotype as revealed by a study involving more than 55,000 individuals completing the Munich ChronoType Questionnaire (Roenneberg et al., 2007). Peak activity timing can differ between individuals for as much as 10 h, as shown in patients with metastatic colorectal cancer (Innominato et al., 2014). The variation in interindividual pharmacokinetics further complicate the selection of dosing time by altering the time within the therapeutic window, and may affect the evaluation of circadian differences in tolerability (Innominato et al., 2010). The interindividual variations indicate that a single best dosing time concluded from large-scale clinical trials rarely fits everyone and underscores the need for personalisation (i.e. to anchor drug administration time to individual circadian oscillations). Implementing chronotherapy also poses substantial practical challenges. For patients, particularly those already taking multiple medications or managing complex conditions where adherence is already challenging, altering dosing times may further increase confusion and reduce compliance; limited patient interest or understanding of circadian-based strategies may present an additional barrier (Kaur et al., 2016). For healthcare professionals, additional workload pressures may limit their ability to carry out individualised chronotherapy (Kaur et al., 2016). The challenges on the healthcare side can be especially evident in high-acuity settings, such as intense-care units, the costs of circadian assessment tools and dynamic lighting systems may be prohibitive, and institutional priorities and culture can further influence feasibility, as outlined in a statement by the American Thoracic Society (Knauert et al., 2023) and a recent work by Hiemstra et al. (2025). Here, the combination of wearable technology to track individual rhythms, coupled with prompting may offer an attractive technological solution.

### Lifestyle interventions

Non-pharmacological interventions provide a powerful complement to medications when taking the environment into consideration. Behavioural approaches, such as maintaining regular daily schedules, optimising light exposure and aligning food intake with the natural light-dark cycle, help resynchronise body clocks with the external environment and, thereby, restore systemic clock function (Sulli et al., 2018; Jacob et al., 2020). These approaches may be particularly suitable for the multi-organ dysfunction in chronic inflammatory diseases, as they restore the inherent regulatory property of the circadian clock and improve overall health outcomes beyond inflammation, without the need to target each comorbidity individually. Clinically, interest in such lifestyle-based circadian interventions has expanded rapidly in recent years. Time-restricted eating aimed at modifying dietary circadian rhythms has shown remarkable benefits regarding restoration of immune-metabolic homeostasis and reduced risk or progression of conditions, such as cardiovascular disease, non-alcoholic steatohepatitis and fibrosis (Palomar-Cros et al., 2023; Gallage et al., 2024). Evidence from animal studies further shows that time-restricted feeding can prevent obesity and metabolic syndrome in mice (Chaix et al., 2019). Notably, even when mice lack their internal behavioural rhythms due to genetic knockout of clock components, imposing a feeding-fasting rhythm still helps maintain metabolic homeostasis (Chaix et al., 2019). Restoring intestinal clock functions by restricted time feeding improves IBD symptoms in mice (Niu et al., 2024). Overall, recognising and addressing how environmental and behavioural rhythms influence the onset and progression of chronic inflammatory diseases represents a crucial, yet still underexplored, direction for future research.

## Future directions

Circadian biology has remarkably reshaped our understanding of chronic inflammatory diseases, offering fresh perspectives on longstanding mechanistic questions and therapeutic challenges. Meanwhile, the rapidly advancing field also opens new frontiers for exploration. Looking ahead, we identify several major directions that may guide the next phase of research. First, for basic research, to delineate how the circadian clock integrates immune and metabolic networks across molecular and systemic levels. Second, for circadian medicine, to utilise the endogenous capacity of the circadian clock to restrain inflammation and restore the whole-body immune−metabolic balance. Third, to further integrate environmental and life-style factors into the current framework of knowledge and therapeutics.

### Basic research

At basic research level, a key step is to further elucidate the molecular pathways that link the core circadian machinery to manifestations of chronic inflammatory diseases. This research direction is now greatly facilitated by advances in high-throughput, single-cell and multi-omics technologies that allow the examination of biological regulation at unprecedented scale and resolution. These tools allow us to probe how the circadian clock components act at the chromatin level, how circadian transcription and modification integrate with cytokine and metabolite signalling, and how these interactions are coordinated across different cell types (Otobe et al., 2026; Nikhil et al., 2025; Yamashita et al., 2026). Mapping this regulatory architecture carries great practical value, as it helps identify nodal targets within the multi-layered clock-immune-metabolic network that are most amenable to precise therapeutic modulation.

Equally important is to bridge these molecular insights with organ- and organism-level physiology. Chronic inflammatory diseases often manifest as whole-body disturbances; yet much of the recent progress in this field of research has focused on mechanisms in isolated cells or tissues, as in examples discussed above. While such approaches have made remarkable contributions in revealing mechanistic understanding, it is now timely to return to the systemic perspective to investigate how local rhythmic processes integrate across organs to sustain homeostasis or drive multimorbidity. An important question is how perturbations acting through circadian, immune or metabolic inputs differentially engage the largely overlapping signalling network to produce distinct outcomes. Ultimately, bridging molecular and systemic understanding is essential to disentangle the causal relationships and relative contributions of circadian, immune and metabolic dysregulation across different types of chronic inflammatory disease.

### Circadian-based interventions

Conventional management of chronic inflammatory diseases often relies on multiple medications, particularly in patients with multimorbidity (Sav et al., 2015). This imposes a substantial treatment burden, encompassing cumulative side effects, financial costs, time demands and psychosocial strain, and increasing demand on healthcare systems (Sav et al., 2015). To reduce this burden and better align medical care with the realities of daily life, to simply refine or multiply conventional medications targeting individual pathological endpoints may no longer suffice. Instead, a paradigm shift is needed towards strategies that restore the intrinsic capacity of the body for self-regulation and homeostatic balance.

Harnessing endogenous regulatory systems represents a powerful, yet underexplored approach to achieving this goal, and circadian-based interventions stand out as a promising avenue. They differ from conventional pharmacotherapy in that they target circadian timing mechanisms that orchestrate metabolic, immune and behavioural processes across virtually all tissues. By realigning or reestablishing internal circadian timings, circadian therapies may simultaneously mitigate multiple disease symptoms, enhance treatment efficacy and reinforce the resilience of the body to pathological perturbations, potentially reducing the number of required drugs and associated adverse effects.

Translating circadian concepts into clinical practice will involve personalised circadian medicine, in which therapeutic timing and dosing are adapted to the chronotype and lifestyle of an individual, rather than relying solely on population averages. Likewise, clinical trial design could also be refined by anchoring intervention schedules to the endogenous circadian phases of  participants, inferred from physiological, behavioural (i.e. sleep−wake, meal timings) or molecular markers. To assess individual circadian rhythms, commonly used methods include measuring plasma or saliva melatonin levels, recording rest-activity rhythms and body temperature via wearable devices, keeping a daily sleep log, and chronotype and alertness questionnaires (Reid, 2019). New approaches measuring transcriptomic or metabolomic biomarkers look achievable, at least for clinical trial application (Wittenbrink et al., 2018). Methods integrating these parameters would allow for more-comprehensive assessment and easier application in daily life and clinics. For instance, non-invasive platforms enable real-time assessment of individual circadian rhythms and prediction of optimal activity or treatment windows (Dose et al., 2023). Continued collaboration between academia, industry and clinics is essential to integrate circadian-informed interventions and tools into mainstream care, and to advance this broader vision of restoring intrinsic homeostasis as a cornerstone of next-generation therapeutics.

### Integrating lifestyle and environmental factors

Most chronic inflammatory diseases have substantial environmental and lifestyle underpinnings, including disrupted daily schedules, unbalanced diets, infections or allergen exposures and chronic psychosocial stress (Furman et al., 2019). Consequently, there are inherent limits to how much genetic analysis alone can explain disease mechanisms and, equally, how far pharmacological interventions can succeed if these environmental drivers remain unaddressed. Effective prevention and treatment, therefore, require looking beyond the molecular level to the context in which disease arises: the environmental and behavioural milieu that sustains pathological processes.

As discussed above, many lifestyle-based circadian interventions already embody this perspective to some extent. To advance this field, it is essential to investigate the combined effects of co-occurring lifestyle and environmental factors in real-world settings, such as the interaction between shift work and high-fat diets. Among the many contributing factors, identifying those that are modifiable is critical for the development of effective preventive and therapeutic strategies. Non-pharmacological approaches that prioritise correcting environmental drivers, both before disease onset and during existing conditions, are essential for relieving the burden of chronic inflammatory diseases on healthcare systems worldwide.

One approach in modern medicine that aligns well with this goal is the concept of the exposome, which is defined as “life-course environmental exposures, including lifestyle factors” (Wild, 2005). The exposome encompasses not only behaviours, such as diet, occupation and sleep−wake patterns, but also broader psychosocial and socioeconomic contexts that collectively influence disease development. In this framework, biomarkers traditionally associated with disease outcomes (e.g. markers of inflammation and oxidative stress) can also be interpreted as markers of modifiable exposures, providing valuable insights for disease prevention and treatment (Wild, 2025).

However, significant challenges still exist. The field still lacks standardized methodologies for assessing and integrating environmental and circadian factors across different studies. From a practical standpoint, large-scale implementation may be constrained by resources and costs (Kunikullaya, 2025). Overcoming these limitations will require ongoing, interdisciplinary collaboration among researchers, clinicians and policymakers. Integrating environmental and behavioural factors alongside genetic and molecular aspects holds the potential to transform the understanding and management of chronic inflammatory diseases, shifting the focus from disease control to the active preservation and restoration of health.

In conclusion, tackling chronic inflammatory diseases requires a comprehensive strategy that integrates three interconnected dimensions: molecular precision, intrinsic homeostatic regulation and holistic environmental modification. Elucidating the molecular connections between the clock mechanisms and chronic inflammation of the human body is crucial for identifying new drug targets and optimising the timing of existing therapies. Personalised circadian medicine, together with behavioural interventions, can help restore the intrinsic capacity of the body for homeostasis and physiological balance. Identifying and correcting modifiable environmental and lifestyle factors further shifts the focus from reactive disease management towards proactive health maintenance and disease prevention. As these dimensions converge, future healthcare can evolve from treating isolated symptoms to strengthening intrinsic resilience of the body to chronic perturbations. This integrated vision defines the next generation of precision and preventive medicine, in which molecular insight, personalised timing and environmental alignment work together to sustain long-term health, and reduce the global burden of chronic inflammatory diseases.
